# Anti-salivary gland protein 1 antibodies in two patients with Sjogren’s syndrome: two case reports

**DOI:** 10.1186/1752-1947-8-145

**Published:** 2014-05-11

**Authors:** Sahana Vishwanath, Long Shen, Lakshmanan Suresh, Julian L Ambrus

**Affiliations:** 1 Department of Medicine, SUNY at Buffalo School of Medicine, 100 High Street, Buffalo, NY 14203, USA; 2 Department of Oral Diagnostic Sciences, SUNY at Buffalo School of Dental Medicine & Immco Diagnostics, 60 Pineview Drive, Buffalo, NY 14228, USA; 3 Buffalo General Hospital, Room C281, 100 High Street, Buffalo, NY 14203, USA

## Abstract

**Introduction:**

Current diagnostic criteria for Sjogren’s syndrome developed by the American College of Rheumatology include the presence of antinuclear antibodies, rheumatoid factor, anti-Ro or anti-La autoantibodies. The purpose of this report is to describe two patients with biopsy-proven Sjogren’s syndrome lacking these autoantibodies but identified by antibodies to salivary gland protein 1. Diagnosis was delayed until salivary gland tumors developed in these patients because of the lack of the classic autoantibodies. This report emphasizes the existence of patients with primary Sjogren’s syndrome who lack autoantibodies anti-Ro or anti-La and may therefore be misdiagnosed. Antibodies to salivary gland protein 1 identify some of these patients.

**Case presentation:**

Two patients are described and were seen in the autoimmune disease clinics of the State University of New York (SUNY) at the Buffalo School of Medicine. In both patients, chronic dry mouth and dry eye had been dismissed as idiopathic because test results for autoantibodies anti-Ro and anti-La were negative. Both patients had swelling of major salivary glands that prompted biopsies. Biopsies of major salivary glands from both cases demonstrated salivary gland tumors and existence of inflammation consistent with Sjogren’s syndrome. Serologic testing revealed antibodies to salivary gland protein 1.

**Conclusions:**

Patients presenting with classic clinical symptoms of dry mouth and eyes do not always show the current serologic markers of Sjogren’s syndrome, anti-Ro and anti-La. In these cases, investigation for antibodies to salivary gland protein 1 is of importance to make the diagnosis of Sjogren’s syndrome. Early diagnosis of Sjogren’s syndrome is necessary for improved management as well as for vigilance regarding potential complications, such as salivary gland tumors as were seen in the described cases.

## Introduction

Sjogren’s syndrome (SS) is an autoimmune disease that starts in the salivary and lachrymal gland but eventually involves multiple other organs including the lungs, kidneys and nervous system. It exists in 0.5 to 3 percent of the population, but as many as 75 percent of the patients may be missed because either they do not seek medical attention and/or their physicians do not look for it. Furthermore, many patients may not meet all the current diagnostic criteria. The most serious complication of SS is salivary gland and gastrointestinal tumors, most commonly B cell lymphomas, which develop in approximately 5 percent of patients. The current diagnostic criteria for SS from the American College of Rheumatology requires three of four objective criteria or four of six total criteria that include: (1) ocular symptoms, (2) oral symptoms, (3) ocular signs, (4) focal sialoadenitis, (5) salivary gland involvement, (6) antinuclear antibodies (ANA), rheumatoid factor (RF), anti-Ro or anti-La autoantibodies in the absence of head and neck radiation treatment, hepatitis C, acquired immunodeficiency syndrome (AIDS), sarcoidosis, graft versus host disease or anticholinergic drugs [1]. Diagnostic criteria are currently being reassessed as they tend to miss the patients with early disease who are most amenable to beneficial therapies.

## Case presentations

### Case 1

A 60-year-old Caucasian woman presented to our Rheumatology Clinic with complaints of persistent dry mouth, dry eyes and a painful swelling of the left side of her neck. Some dry mouth and dry eyes had been present for 18 years but the more severe symptoms developed over the period of six months prior to being seen. Periodic serology studies for ANA, anti-Ro and anti-La were all negative. Had she been assessed for SS, she would have met only two criteria for SS based on ocular symptoms and ocular signs. She developed swelling on the left side of her neck that prompted her to visit her physician. At that time, the evaluating physician noted a swollen submandibular gland and cervical lymphadenopathy. She had a partial submandibular gland resection along with an excisional biopsy of the lymph node, which was then determined to be ‘nonspecific inflammation’. Her symptoms of dryness of mouth persisted and she developed dryness of her eyes requiring the use of artificial tears six years before her presentation to our clinic. She had no comorbidities at the time of her initial presentation. The family history was notable for a sister with celiac sprue and antiphospholipid antibodies.

A clinical examination showed dry eyes, dry mouth and mild swelling of the left submandibular and parotid glands. Her laboratory evaluation included a normal complete blood count, comprehensive metabolic profile and urinalysis. ANA, anti-Ro and anti-La test results were all negative. A lip biopsy was also performed and showed nonspecific inflammation of the minor salivary glands. Additional evaluations revealed the presence of antiphospholipid antibodies and antibodies to salivary gland protein 1 (Sp1).

**Figure 1:**
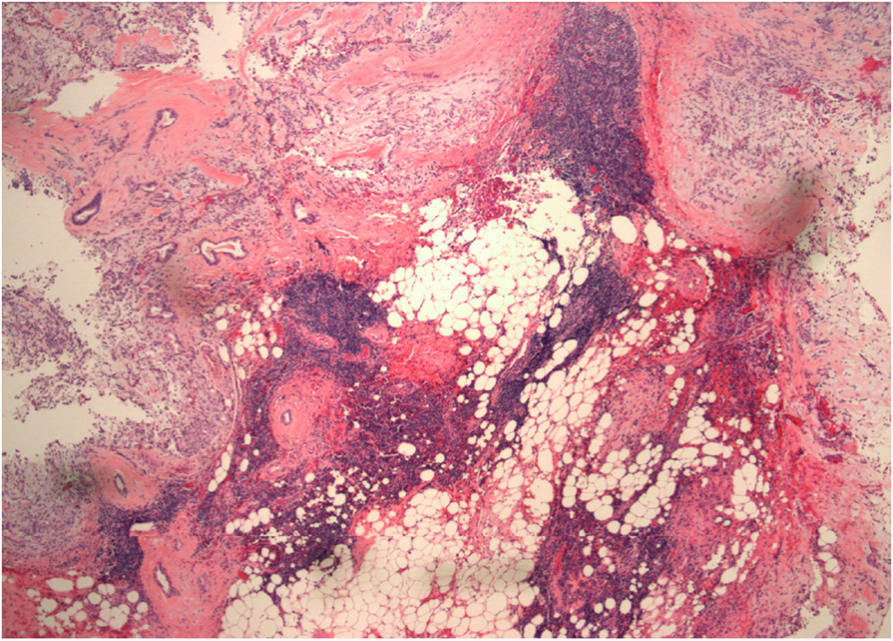
Pleomorphic adenoma in the parotid gland with multiple lymphocytic foci in the surrounding tissue consistent with Sjogren’s syndrome (hematoxylin and eosin, original magnification 4×).

Our patient had subsequent resection of the left parotid gland. The histology showed pleomorphic adenoma of the left parotid gland and lymphocyte-predominant inflammation, consistent with Sjogren’s syndrome (Figure 1).

### Case 2

**Figure 2:**
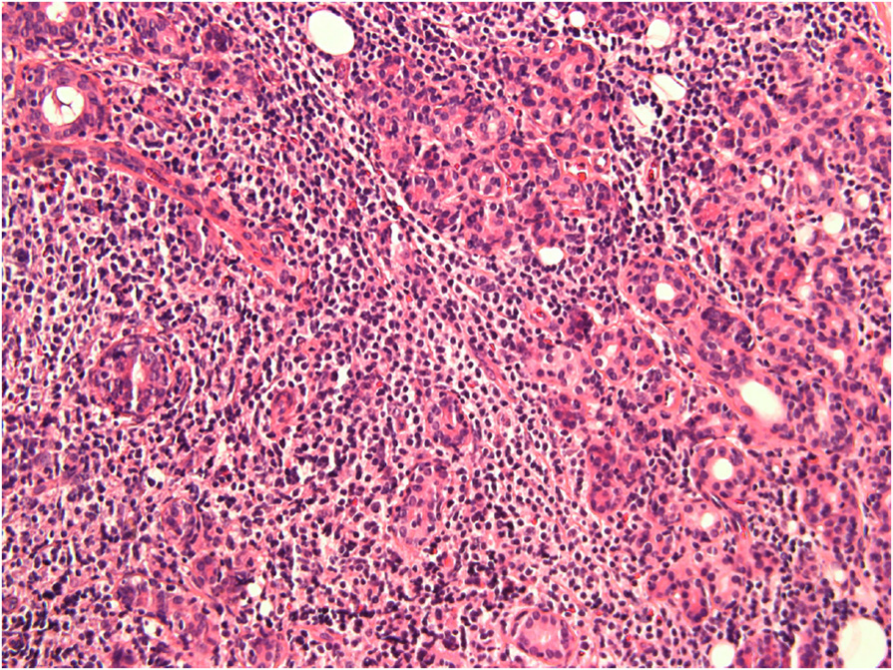
Lymphocytic proliferation in the parotid gland affecting the glandular elements (hematoxylin and eosin, original magnification 40×).

A 63-year-old Caucasian woman presented to our Rheumatology Clinic with complaints of persistent dry mouth, dry eyes and swelling on the right side of her eye. The swelling had been present for three months. She had had a four-year history of dry eyes and dry mouth. Her primary physician ordered anti-Ro and anti-La antibody tests on two occasions, both of which were negative. She would have met two criteria for SS at this time, ocular symptoms and oral symptoms. She was treated with artificial tears and oral liquids. After two years, she developed swelling in her right orbital region and progressive droopiness of her right eyelid. An excision biopsy of this mass showed non-Hodgkin lymphoma of the lachrimal gland. She subsequently developed swelling of both of her parotid glands, her left more than her right. A positron emission tomography (PET) scan was done that revealed increased uptake in both parotid and submandibular glands. She underwent an excisional biopsy of the left parotid gland. Histological evaluation of the tissue revealed a marginal zone lymphoma that was CD20+, CD23+ and CD10-. Areas of lymphocytic infiltration were noted consistent with a diagnosis of SS that prompted the consultation in our clinic (Figure 2).

On presentation to our clinic, she was noted to also have a past medical history of paroxysmal nocturnal hemoglobinuria, hepatitis C secondary to multiple blood transfusions, hypoparathyroidism, celiac sprue, interstitial cystitis, Raynaud’s phenomenon and three previous miscarriages. Previous workup for antiphospholipid antibodies was negative. Our patient was referred to us for postsurgical follow-up and management. On physical evaluation, she was noted to have dry mouth, livedo reticularis and postsurgical scars. Her laboratory evaluation in our clinic included tests for ANA, anti-Ro and anti-La that were negative. Antibody testing for Sp1 was performed and was positive.

## Discussion

Sjogren’s syndrome is a systemic autoimmune disease starting in the salivary and lachrymal glands but eventually including involvement of multiple other organs. SS can also occur secondary to other autoimmune diseases, such as lupus, and rheumatoid arthritis [2]. Patients with SS, typically present with a dry, gritty sensation in the eyes and dry mouth. At this stage, there has already been significant destruction of the salivary and lachrymal glands. There is in fact a high frequency of SS in patients with dry eyes that is frequently missed [3,4]. Typically, involvement of the lachrymal and submandibular glands occurs before involvement of the parotid glands. Destruction of the sublingual glands is unusual. Lung and kidney disease tend to occur late in the disease process. As many as 5 percent of patients with SS develop B cell lymphoma, most commonly occurring in the salivary glands and gastrointestinal tract [5]. One of our patients developed B cell lymphoma.

Many investigators utilize diagnostic criteria for SS based on the revised American-European Consensus Group which include (1) ocular symptoms, (2) oral symptoms, (3) ocular signs, (4) focal sialoadenitis, (5) salivary gland involvement, (6) anti-Ro/La antibodies in the absence of head and neck radiation treatment, hepatitis C, AIDS, pre-existing lymphoma, sarcoidosis, graft versus host disease or anticholinergic drugs. Diagnosis is based on meeting three of four objective criteria or four of six total criteria [6]. The recent American College of Rheumatology classification criteria for diagnosis of SS also includes ANA and RF along with anti-Ro and/or anti-La autoantibodies. The only objective findings included are labial salivary gland biopsy exhibiting focal lymphocytic sialoadenitis with a focus score ≥1 focus/4 mm^2^ and keratoconjunctivitis sicca with an ocular staining score ≥3 [1]. Our patients lacked ANA, RF and anti-Ro or anti-La antibodies and were minor salivary gland (lip) biopsy negative. They would not have fulfilled the diagnostic criteria of SS on presentation.

The autoantibodies that are most commonly believed to be associated with SS are anti-Ro and anti-La. However, there are multiple other antibodies that are being studied including antibodies to muscarinic receptor 3, tissue kallikrein, alpha-fodrin, carbonic anhydrase II and VI, parotid-specific protein and Sp1. The significance of these autoantibodies has not been fully appreciated [7].

As SS is typically identified at later stages, it has been difficult to study the early events occurring in humans. An animal model that faithfully reproduces the features of SS seen in patients, the interleukin 14 alpha transgenic (IL14αTG) mouse, has been used to study early events in the disease [8-12]. The use of this animal model led to the discovery of the autoantibodies to Sp1, carbonic anhydrase VI (CA6) and parotid secretory protein (PSP). The presence of these autoantibodies in the sera of patients with SS has been confirmed [11]. As per these studies, only 25 percent of the IL14αTG mice developed anti-Ro/La antibodies. Interestingly, the time course in the development of the autoantibodies showed that the Sp1 and CA6 antibodies were present very early in the disease, while antibodies to Ro/La occurred late in the disease [11]. In unpublished studies, antibodies to Sp1 and CA6 were found in less than 5 percent of normal controls and patients with rheumatoid arthritis lacking secondary SS [13].

The clinical presentation of both our patients was very typical for SS. Our first patient initially had symptoms of dry mouth and dry eyes, then had submandibular gland involvement, followed by parotid gland involvement. She ultimately developed pleomorphic adenoma, a benign salivary gland tumor. Our second patient had dry eyes and dry mouth for multiple years followed by lymphoma of the lachrymal and parotid glands. Neither patient had antibodies anti-Ro or anti-La, nor did their minor salivary gland biopsies show classic features of SS. Only the development of tumors in these cases necessitated major salivary gland resections. Both the major salivary gland tissues showed classic histological features of SS in addition to the tumors.

These cases stress the importance of using autoantibodies besides anti-Ro and anti-La in the diagnosis of SS. In these patients, the presence of anti-Sp1 antibody helped in the diagnosis of SS. We did not have early sera to evaluate so we cannot determine when in the time course of these patients the antibodies to Sp1 occurred, but we suspect that they should have been present at an early time point in the disease. The presence of these antibodies was noted even before the presence of lymphocytic infiltration of the gland in the animal model for SS that we utilized [11]. There are likely many patients with SS who lack autoantibodies anti-Ro or anti-La. Investigation for antibodies to Sp1 and other markers such as CA6 and PSP will help in the diagnosis of the patients.

The current management of SS is mostly symptomatic. By the time of diagnosis, there is almost complete destruction of the salivary glands. The earlier detection of SS with these autoantibodies may allow the development of new forms of therapy that can preserve the function of the salivary and lachrymal glands.

## Conclusions

These cases illustrate the existence of a novel autoantibody, anti-salivary gland protein (Sp1), which identifies certain patients with SS who lack autoantibodies anti-Ro or anti-La. In each case, dry eyes and dry mouth suggested the possibility of SS that was discounted because of the lack of anti-Ro or anti-La. Malignancy in the salivary glands led to biopsies that demonstrated features of SS. Both patients had antibodies to Sp1. Patients with features of SS who lack antibodies anti-Ro or anti-La should be evaluated for antibodies to Sp1. Early identification of patients with SS will ultimately lead to better therapies that will hopefully prevent the development of salivary gland malignancies.

## Consent

Written informed consent was obtained from both patients for publication of this case report and any accompanying images. Copies of the written consents are available for review by the Editor-in-Chief of this journal.

## Abbreviations

ANA: Antinuclear antibodies; CA6: Carbonic anhydrase VI; PSP: Parotid secretory protein; RF: Rheumatoid factor; Sp1: Salivary gland protein 1; SS: Sjogren’s syndrome.

## Competing interests

The assays for Sp1, CA 6 and PSP are licensed by SUNY at Buffalo to Immco Diagnostics and are commercially available through Immco Diagnostics. Julian L. Ambrus Jr., MD and Long Shen, PhD are entitled to royalties under this agreement and Lakshmanan Suresh, DDS, MS is an employee of Immco Diagnostics.

## Authors’ contributions

SV assisted in the care of the patients. LS assisted in the performance of the assays for Sp1. LS evaluated the histology of the salivary glands and assisted in the assays for ANA, RF, anti-Ro, anti-La and anti-Sp1 and participated in the writing of the manuscript. JA took care of the described patients and reviewed all the data. All the authors participated in the writing of the manuscript and read and approved the final manuscript.
